# System for quantitative evaluation of DAB&H-stained breast cancer biopsy digital images (CHISEL)

**DOI:** 10.1038/s41598-021-88611-y

**Published:** 2021-04-29

**Authors:** Lukasz Roszkowiak, Anna Korzynska, Krzysztof Siemion, Jakub Zak, Dorota Pijanowska, Ramon Bosch, Marylene Lejeune, Carlos Lopez

**Affiliations:** 1 Nalecz Institute of Biocybernetics and Biomedical Engineering Polish Academy of Sciences, Ks. Trojdena 4 st., 02-109 Warsaw, Poland; 2 Pathology Department, Hospital de Tortosa Verge de la Cinta, Institut d’Investigacio Sanitaria Pere Virgili (IISPV), URV, Tortosa, Spain; 3 Molecular Biology and Research Section, Hospital de Tortosa Verge de la Cinta, Institut d’Investigacio Sanitaria Pere Virgili (IISPV), URV, Tortosa, Spain; 4 Medical Pathomorphology Department, Medical University of Bialystok, Białystok, Poland

**Keywords:** Imaging, Microscopy, Immunohistochemistry, Cancer, Biomedical engineering

## Abstract

This study presents CHISEL (Computer-assisted Histopathological Image Segmentation and EvaLuation), an end-to-end system capable of quantitative evaluation of benign and malignant (breast cancer) digitized tissue samples with immunohistochemical nuclear staining of various intensity and diverse compactness. It stands out with the proposed seamless segmentation based on regions of interest cropping as well as the explicit step of nuclei cluster splitting followed by a boundary refinement. The system utilizes machine learning and recursive local processing to eliminate distorted (inaccurate) outlines. The method was validated using two labeled datasets which proved the relevance of the achieved results. The evaluation was based on the IISPV dataset of tissue from biopsy of breast cancer patients, with markers of T cells, along with Warwick Beta Cell Dataset of DAB&H-stained tissue from postmortem diabetes patients. Based on the comparison of the ground truth with the results of the detected and classified objects, we conclude that the proposed method can achieve better or similar results as the state-of-the-art methods. This system deals with the complex problem of nuclei quantification in digitalized images of immunohistochemically stained tissue sections, achieving best results for DAB&H-stained breast cancer tissue samples. Our method has been prepared with user-friendly graphical interface and was optimized to fully utilize the available computing power, while being accessible to users with fewer resources than needed by deep learning techniques.

## Introduction

The pathologists evaluate a wide range of specimens obtained from surgical procedures based on the histopathological tissue sections. To aid in the diagnosis, prognosis and prediction, particular staining techniques, such as immunohistochemical (IHC) staining, are performed. Examination of tissue sections is typically carried out by an experienced pathologist directly by the use of a microscope. However, the application of digital sample images has become more common with the rapid growth of digital pathology and the availability of whole-slide imaging (WSI) systems.

The evaluation performed by a human expert is inherently irreproducible as neither the person nor the algorithm can redo it in exactly the same way. The time spent by a specialist looking at a glass slide and making a regular diagnostic decision might be very short for a typical sample. However, in the case of quantitative analysis, the specificity of this task makes it very time-consuming and prone to errors. This type of analysis allows identifying the structure or pattern of cell localization which in turn can yield important information. Features, such as density, can be used for predicting treatment effectiveness^1,2^. Detection and consequently segmentation of cells or their structures in digital microscopic images of tissue can enable obtaining information about high-quality features for nuclear morphometrics. They may be the basis for the new discoveries in digital pathology.

Over the years, multiple computer-aided image processing methods have been proposed as an alternative to this laborious task. Yet, the processing of IHC-stained tissue samples is still not fully satisfactory and remains a challenging scientific problem. In comparison to the often used hematoxylin and eosin (H&E) stained specimens, the colors in the IHC-stained samples are less consistent^3–6^, which is considered as a major issue in fully automatic image processing systems. In addition, a multitude of obstacles and limitations prevent both a specialist and an algorithm from achieving a consistent evaluation result, which include: variability in shape, size, and color of the objects of interest; lack of definite criteria for the identification of cell type; lack of defined stain intensity cut-offs; and presence of overlapping structures. A successful image processing approach should overcome all these limitations in a robust way, in order to ensure high quality and accuracy in various situations.

It is estimated that 2.2 millions of new cases were detected and about 685 thousand patients, mainly women, died from breast cancer in 2020^7^. It is the second most common cancers among women that results in death^8^. It has been already proven that a adequate prognosis in breast cancer might be established based on morphological tumor differentiation^9^, but as evaluation is based upon a subjective expert opinion about histologic features. Difficulties in consistency and reproducibility are inevitable. Fast and reliable diagnosis, more insightful prognosis or new understanding of disease mechanism could be established with the help of computer aided evaluation of biopsy digital images. The first step of such evaluation is quantitative analysis, which still poses a challenging problem for the scientific community.

In this study, we describe CHISEL (Computer-assisted Histopathological Image Segmentation and EvaLuation), a system for the quantitative evaluation of digitized IHC stained slides. We propose an end-to-end method which is capable of processing benign and malignant (i.e. breast cancer) tissue samples with nuclear staining of various intensity and diverse cellularity. It has a foundation in the results of previous experiments regarding comparison of effectiveness of adaptive threshold methods and establishing cluster splitting method. This article is focused on: development of the end-to-end solution, elaborating and verification of scheme for analyzing whole-slide tissue images by dividing them into fragments, analysis of parameter optimization and its impact on the performance, and comparison of achieved results with results of other methods.

CHISEL stands out with the proposed seamless segmentation based on regions of interest (ROIs) cropping as well as the explicit step of nuclei cluster splitting followed by a boundary refinement. We compared it with the methods that provide state-of-the-art results and validated its effectiveness using two datasets: (1) manually labeled set of images of breast cancer cells with IHC staining against FOXP3 (forkhead box P3) using 3,3′-diaminobenzidine and hematoxylin (DAB&H)^10^ and (2) publicly available Warwick Beta Cell Dataset^11^ containing images of IHC-stained pancreas tissue sections with provided ground truth.

The source code is available on https://github.com/knave88/CHISEL/ and project’s webpage https://www.ibib.waw.pl/en/scientific-activity/projects/167-umo-2013-11-n-st7-02797/233-chisel.

### Related works

Many different techniques have been proposed for the quantification of cell nuclei in the digital images of tissue sections. Nuclei are most often detected using the global or local threshold method as presented in a review^12^ on H&E-stained histologic slides. It is a simple technique involving low computational complexity. Reducing the computational complexity is extremely important in case of enormous digital images of tissue samples scanned at a high resolution. Thresholding is generally applied to monochromatic images; therefore, it is necessary to perform extraction, deconvolution, or conversion to different color models, such as original RGB^13^, color deconvolution^14–17^, HSV^18,19^, and CIELab^20–22^ as a prior step.

To accurately segment the nuclei, apart from the threshold-based techniques, numerous other methods have been proposed based on geometric active contour^23^, feature collection^21^, level-set^24^, clustering^22,25^ and SVM^17,26^.

Moreover, new approaches are available with deep learning (DL) and convolutional neural networks for nuclei quantification^12,27–30^. In histopathology, the application of DL is relatively new^31^. The majority of published papers have focused on the detection of mitotic figures presented during mitosis^32–36^ or tissue classification for various types of cancer^37–41^.

Studies related to IHC staining are sparse. Xie et al.^42^ described a very interesting approach to IHC staining based on block processing of images. More recently, Pan et al. proposed a method for nuclei segmentation based on the deep semantic network, which can give promising results in multiorgan tissue samples^43^.

The best part of publications of novel methods suggested for the detection of nuclei or cells involve the use of tissue stained with H&E. Worth mentioning is the work of Cui et al.^44^ that reported an automatic end-to-end deep neural network for the segmentation of individual nuclei in high-resolution histopathological images of tissue obtained from various organs and stained with H&E. Additionally, many researchers have presented novel DL models for nuclei detection in H&E images such as Shape Priors with Convolutional Neural Networks^45^, Spatial Information Convolution Neural Network^46^, and Spatially Constrained Convolutional Neural Network^47^. However, to the best of our knowledge, none of these have released the code applicable for testing and comparison.

### Reference methods

Besides the strictly novel aforementioned scientific reports, some state-of-the-art frameworks for nuclei quantification in tissue samples have been developed and released as freeware, thus they are available for comparative evaluation. Among them, we compared Qupath^48^, IHC toolbox^49^, and Tmarker^50^ with CHISEL in this investigation. Qupath is quite popular in the pathology community as it is universal to the used staining (H&E, IHC), can process whole-slide images, and cooperates with ImageJ software. Tmarker is based mainly on color deconvolution and segmentation with superpixel-based approach. IHC toolbox is an ImageJ plugin that uses oval fitting for nuclei segmentation.

The DL methods are gradually overtaking the classical image processing methods, which deal with feature space division. This is particularly noticeable in the case of images, in which the features of objects are uncertain and variable as it is in the images of stained tissue samples we are dealing with. Hence, we decided to include, in the results comparison, two DL techniques. Unet^51^ is a very commonly used baseline model, and so, in this study, we compare our method with the Unet model trained from scratch on ScienceBowl2018 dataset^52^. The other DL method used for comparison was published online by Chen et al.^53^ as a pretrained model.

Both referenced solutions were run in Python (training and inference on GPU: Nvidia GeForce GTX 850M) with implementations available online.

## Materials and methods

### CHISEL framework

**Figure 1:**
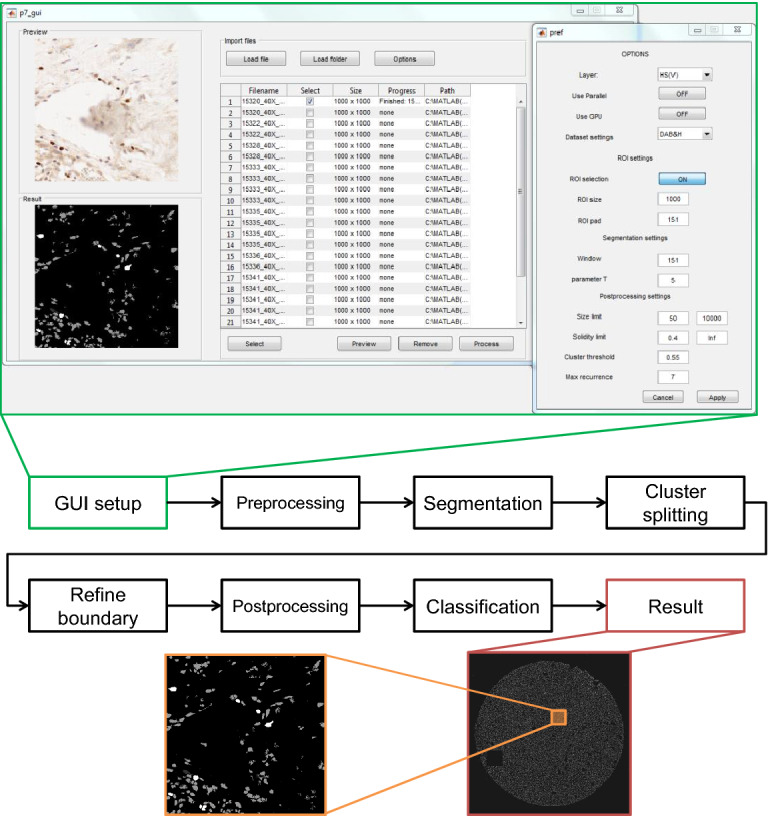
Schema of CHISEL system. GUI is presented above (green lines) with opened preferences submenu where user can modify most critical parameters influencing the processing. Below is presented an example of the resulting image of fragment of TMA (red lines) and its small $1000\times 1000$ pixels zoomed fragment (orange lines) with multiclass output. Source code available on https://github.com/knave88/CHISEL/ [hi-res version of image available online].

In this study, we present a new approach for the segmentation and quantification of nuclei in digital slide images of ICH-stained tissue sections. This method is exceptional due to the proposed seamless segmentation based on ROI cropping as well as the explicit step of nuclei cluster splitting followed by a boundary refinement, as could be seen in schematics in Fig. 1. The framework is capable of processing whole-slide images, tissue microarray (TMA) cores, and samples acquired by the digital camera attached to a microscope.

Tissue samples are prepared in many sizes and shapes, ranging from small cropped images acquired by the digital camera attached to a microscope to enormous whole-slide images acquired with slide scanners. Segmenting vast high-resolution images is a challenging task, mainly due to the limited memory capacity. To comply with the processing capability of the developed system, the images that are too large to fit in the memory are split into smaller ROIs and processed in batches.

**Figure 2:**
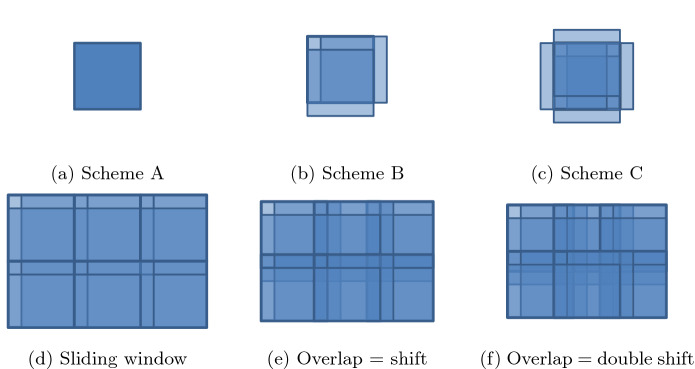
Comparison of three schemes for an overlapped patch extraction (top row). An example of overlap achieved with different stride of sliding window algorithm with Scheme B (bottom row). In (**e**) the example is presented where the stride is balanced so that overlap is equal to the size of the shift in Scheme B. Original ROI (without additional ones) highlighted with thicker lines in every example. More overlapping ROI are denoted with color-darker with more overlapping ROI.

During this investigation, we tested three schemes for an overlapped patch extraction (comparison in Fig. 2). As a primary approach, we extracted patches by sliding window. First, we utilized default MATLAB distinct block processing implementation (*blockproc* function), but in the case of whole-slide images this function was found to be too time-consuming. Therefore, we used it as a benchmark for further development of the method.

*Scheme A.* For the images that are too big to fit in the memory, the framework calculates the locations of all ROIs (with size set by the user; default $1000\times 1000$) which will be used for further processing. Suitable padding is added to the ROI on the border of the images. The ROIs are then cropped out of the original images before further processing. To reduce the number of extracted patches for processing, we have trained the simple fully connected Artificial Neural Network (ANN) to discriminate empty patches and heavily blurred patches, further in this paper referenced as ANN_ROI.

*Schemes B and C.* Similar to Scheme A, the positions of additional shifted ROIs are calculated for every processed ROI. Additional ROIs are shifted in two (right and bottom) directions (Scheme B) or in all four main directions (Scheme C). The shift size is at half the size of adaptive threshold window. Thus, the overlap with the shifted ROIs allows combining information from the overlapping processed fragments of images to achieve precise results on every border of ROI. With stride smaller than the size of ROI much more overlap area is considered, hence the method yields even more accurate final result at the cost of processing time.

The first phase of ROI processing involves color space conversion and extraction of the specified channel for further processing. This approach limits the amount of data to be processed (one channel instead of three). The transformation from RGB to HSV color space enables separating the data consisting of color information from the luminescence intensity information. According to our previous research^54^, processing the Value layer of the HSV model gives satisfactory results together with our subsequent image processing carried out in this workflow.

Next, the Value layer of the HSV color model is subject to the initial segmentation of nuclei. Segmentation is performed with Bradley adaptive threshold algorithm^55^ which is described in detail in an earlier study^54^. This thresholding method makes use of the adaptive cut-off level to discriminate objects from the background. Our previous study proved that this method shows high sensitivity toward both immunopositive and immunonegative nuclei. Then, the results of adaptive thresholding are used as an initial mask of implemented active contour method^56^, performed on Value layer of HSV, in order to further improve segmentation and achieve a boundary that is more closely fitted to the real object. In one study^57^, we established that preliminary object detection followed by localized boundary refinement provides better results than one-step segmentation. Nevertheless, this creates a binary mask with many clustered objects.

Connected objects resulting from the detection phase (thresholding) are a major nuisance in digital pathology as well as in image processing in general. For precise quantification of nuclei, these clustered objects should be separated correctly. A study^58^ showed that recursive local processing provides the best cluster splitting. It is an algorithm based on distance transformation with recurrently increasing threshold value. This method was specifically developed for processing histopathological images and splitting clustered nuclei. Purposely in this method color information is excluded from nuclei separation, which means that only the shape of the cluster is taken into consideration during nuclei separation. Different from typical image processing, in case of digital tissue samples it is an advantage, because of high variability of staining and no defined color cut-off levels.

After cluster splitting, simple postprocessing is performed to get rid of the objects that have an area less than 50 pixels. Such objects are treated as artifacts because at the magnification of 40/20× and image capture resolution of $0.25/0.5\, \upmu \text{m}/pixel$ the objects with area of 50 pixels correspond to $3.1/12.5 \; \upmu \text{m}^2$. The area of nuclei in analyzed datasets was consistently above 300 pixels ($18/75 \; \upmu \text{m}^2$). Consequently with high probability we assumed that the vast majority of objects smaller than 50 pixels cannot be nuclei and are therefore excluded from further processing.

The next step in CHISEL is the merging of multiple processed ROIs to remove ambiguity from the objects located in the overlapping regions. This process increases accuracy in the case of objects that are located near the border of single ROI and partially visible nuclei. For assembling, a majority vote mechanism is applied to classify each pixel. The binary masks resulting from each ROI are added together to create a merged image so that the pixels with higher values are more probable to contain nuclei. For further processing the final mask image is created; the pixels in the overlapped area are labeled as objects, if agreed by the majority of component images.

Next, the obtained binary mask is used to extract the discriminative features of objects, in order to enable classification from the original RGB images based on 103 features extracted from each RGB channel: 11 geometric features, 9 features based on the histogram of oriented gradients, 44 features extracted from the gray level run length matrix, 5 features based on autoregression model, 20 features based on the Haar wavelet, 5 gradient features, and 9 histogram features.

The classification of nuclei, into two classes (immunopositive and immunonegative), is performed with fully connected ANN, that was optimized for this task (more on this in “Optimization” section). The classifier ultimately achieved 99.2% accuracy of classification.

### Method evaluation

Evaluation is performed to determine the number of found objects that are segmented and classified correctly. For method evaluation three metrics were applied: positive predictive value (PPV), true positive rate (TPR), and F1 score. PPV is the ratio of true positives (TP) to the number of detected objects (PPV = TP/(TP + FP)), where FP represents the false positives. TPR indicates the proportion of the objects of interest found (TPR = TP/(TP + FN)), where FN represents the false negatives. F1 score is calculated as the harmonic mean of these metrics. In the case of all metrics, values closer to 1 imply better results.

### Datasets

#### IISPV dataset

The images of breast cancer tissue used for validating the experiments were acquired from the Molecular Biology and Research Section, Hospital de Tortosa Verge de la Cinta, Institut d’Investigacio Sanitaria Pere Virgili (IISPV), URV (Tortosa, Spain) and the Pathology Department of the same hospital. The dataset was formerly acquired during a project^59^ (grant number PI11/0488) conducted at the Instituto de Salud Carlos III (Spain), which was approved by the Ethics Committee of the Hospital Joan XXIII de Tarragona (reference 22p/2011). The subset used in this study was acquired based on international cooperation (Polish National Science Center, grant number 2013/11/N/ST7/02797).

**Figure 3:**
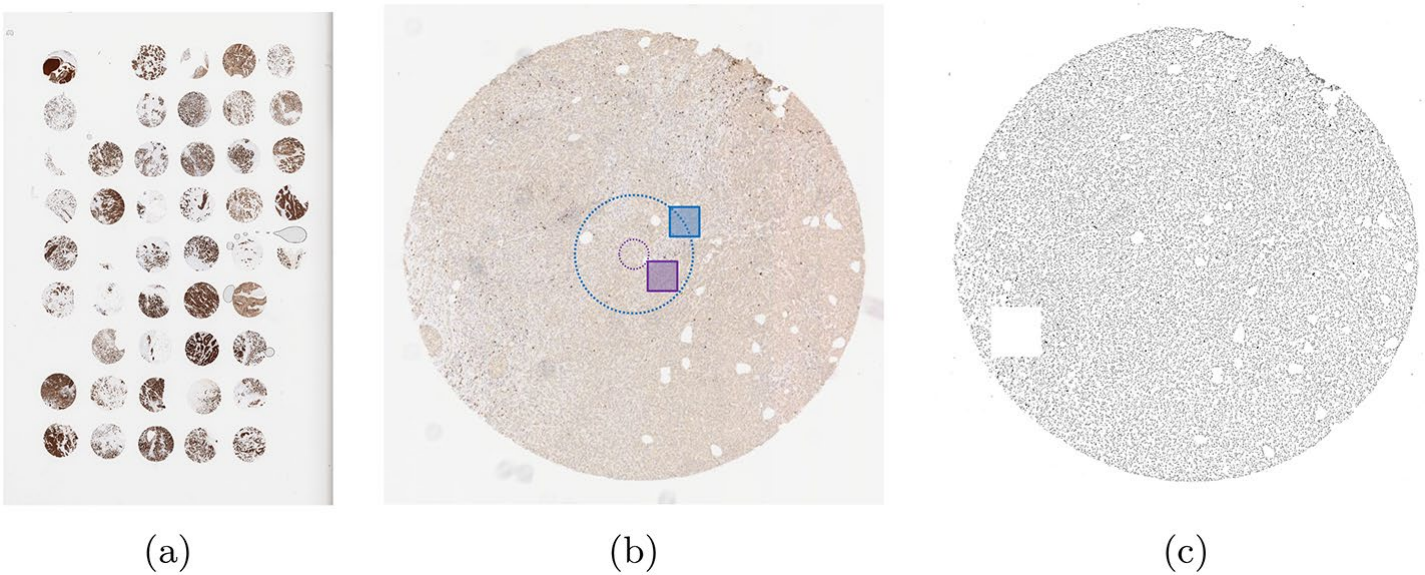
(**a**) Example of TMA with multiple cores processed simultaneously by histochemical processing. (**b**) Example of a single core extracted from the digital image of TMA. ROI selection algorithm pictured with increased size of virtual circles and ROI for better visualization. (**c**) Binary map depicting the seamless detection of nuclei with CHISEL processing on sample presented in the middle column. (Image was inverted for better visual appearance).

The tissue sections of breast and auxiliary node biopsies were subjected to typical histotechnical processing and converted to formalin-fixed, paraffin-embedded tissue blocks, using the procedure detailed in our previous research^59^. TMAs were formed by extracting small cylinders of tissue and arranging them in an array on a prepared paraffin block (example shown in Fig. 3). Subsequently, they were stained using the indirect IHC primary antibodies against FOXP3 and the secondary antibodies, including the peroxidase block, labeled polymer, buffered with substrate/DAB+ chromogen, and finally contrastained with hematoxylin. The simultaneous staining of multiple samples in TMA decreases the intralaboratory variability of staining intensity (differences in staining concentrations between slices). TMAs are often used for studying the patterns of immune response in breast cancer^60–62^.

TMA is also advantageous for processing digital images because multiple samples are acquired together in one scan under the same conditions. Overall, the use of TMA increases the reproducibility of biomarker pattern studies^5^.

Samples were digitalized using the automated whole-slide scanning system (ScanScope, Aperio) with $40\times /0.75$ Plan Apo objective lens. Two designated programs^62,63^ were used to parse the whole-slide images into separate images of TMA cores; example of TMA shown in Fig. 3.

For method evaluation, ROIs in the size of $1000\times 1000$ pixels were randomly extracted from the whole dataset after establishing the selecting algorithm. The upper-left corner of each ROI was drawn from the circumference of two virtual circles (centered with tissue core) with radii of 500 and 2000 pixels, respectively. This consistently provided nonempty images, while maintaining the randomness of samples. In the end, the ROIs were cropped out from the original images for further processing, visualized in (middle part of) Fig. 3.

**Figure 4:**
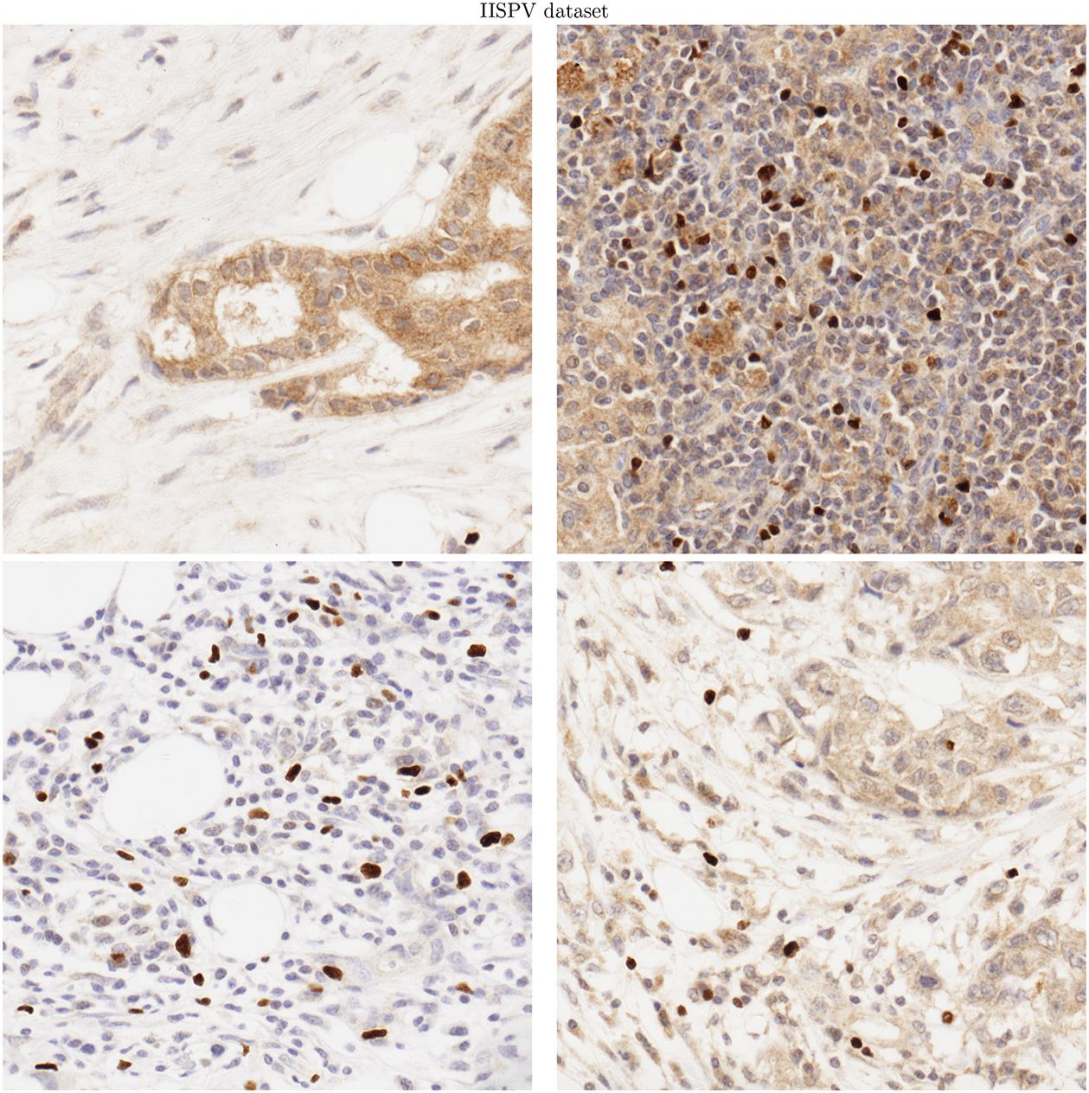
Examples of images from IISPV dataset consisting of DAB&H-stained tissue sections of breast and auxiliary node biopsies, digitized with WSI scanner at ×40 magnification.

The selected images differed in their degree of complexity, architecture compactness, and global contrast, which is typical for this type of biological data (Fig. 4). In addition, they differed in brightness, staining intensity, and colors of structures despite careful standardization of every step of sample processing (tissue preparation, simultaneous staining, and slide digitalization by the same WSI scanner).

Finally, 13 images were manually annotated by two experts. Each expert marked all the locations of the positive and negative nuclei with an indicator for object-wise evaluation. A total of 7557 nuclei markers were present in the ground truth binary images. Markers pinpointed location of nuclei in the image, but were not established as the centroid of the object.

Moreover, as many as 808 nuclei boundaries (405 immunopositive and 403 immunonegative) were labeled. This created the ground truth for pixel-wise evaluation. The ground truth was generated by merging the annotations, it was limited to the area marked by both experts. In each image, all the immunopositive and 31 randomly selected immunonegative objects were annotated with boundary, which resulted in a binary mask.

#### Warwick beta cell dataset (WBCD)

Our proposed system focused on processing the IHC (especially DAB&H)-stained images. Datasets of such images are available online in very few numbers. One of them is Warwick Beta Cell Dataset which provides the ground truth for detecting nuclei of specific cell types examined in the postmortem of diabetes patients. Hence, we decided to test the performance of CHISEL on this dataset.

This dataset was developed to estimate beta cell mass and estimate the number of nuclei within “the insulin-stained area” as it provides valuable information^11^. The dataset consists of 20 images of DAB&H-stained tissue samples. In addition to the images, the ground truth in the form of binary images with point markers locating the beta cells is provided. The markings were made by three independent experts. The three separate markings are given along with their fused version.

Originally, this dataset was used for development of Lipsym^11^ method that was compared with LoG method in original publication. These results are presented as is in Table 2 and compared with CHISEL framework.

In this study, we used 18 images for evaluation, while the two remaining images were used for training the classifier part of CHISEL (described later in “Optimization” section ) to discriminate beta cells. We used the fused version of ground truth for evaluation.

#### Comparison of datasets

Comparing two datasets it is quite easy to observe relatively different features of these images. Firstly, the brown staining in IISPV is located mostly within nuclei while in WBCD images the brown deposit could be seen marking whole areas instead (the beta cells to be quantified are within these stained areas). Although, it is worth pointing out that the nuclei in both datasets significantly stand out from the surroundings with higher intensities. Apart from the difference in stain distribution the properties of the nuclei to be found are dissimilar. Nuclei in IISPV have size of $311\pm 35$ pixels (and $95\pm 9$ pixels perimeter), while WBCD shows nuclei of $144\pm 11$ pixels in size (and $53\pm 6$ pixels perimeter). In general, nuclei present in IISPV dataset are roughly double in size compared to those from WBCD. While it is not crucial for proposed method which is designed to handle microscopic images at magnification of 40× and 20×. What is more, the circularity of nuclei in WBCD is significantly greater than in IISPV dataset, being $0.70\pm 0.08$ and $0.50\pm 0.07$ respectively.

### Ethics declarations

No participants were involved in this study. The data used has been obtained from literature and open sources. “Warwick Beta Cell dataset of H+DAB images of beta cells in mouse pancreatic tissue (released with LIPSyM paper^11^ published in J Pathology Informatics, Jan 2012)” (https://warwick.ac.uk/fac/cross_fac/tia/data/) IISPV dataset was acquired during a project (grant number PI11/0488) conducted at the Instituto de Salud Carlos III (Spain), which was approved by the Ethics Committee of the Hospital Joan XXIII de Tarragona (reference 22p/2011).

## Discussion and results

### Optimization

**Figure 5:**
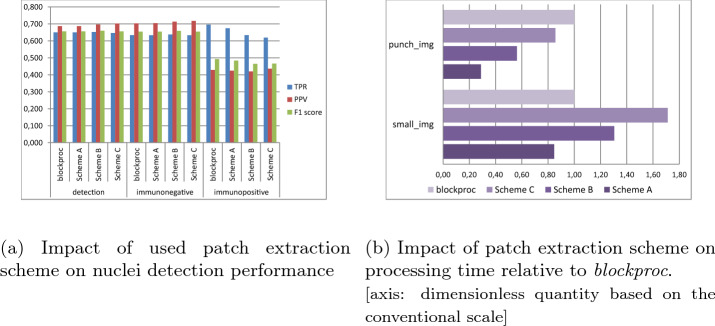
Comparative results of patch extraction schemes proposed in this study. (left) The comparison of detection performance metrics. (right) Time estimation relative to *blockproc* method. Two sizes of images were tested: *punch_img* (one core of TMA) of size $16{,}000\times 13{,}000$ pixels and *small_img* (relatively small cropped image) of size $1000\times 1000$ pixels.

We tested four schemes for patch extraction. The schemes were compared in terms of their effectiveness and time consumption. The efficiency evaluation was based on the metrics TPR, PPV, and F1 score (Fig. 5, left), and the experiment was conducted with IISPV dataset with the size of ROIs set to 250 pixels. Typically, ROIs should be larger but the size of annotated images required reducing their size. The ANN_ROI used in Schemes A, B, and C was trained on 40,000 annotated ROIs and showed 74.3% accuracy in discriminating heavily blurred and empty ROIs.

For time evaluation, we treated the *blockproc* implementation as a reference method, and therefore, all the presented results are relative (Fig. 5, right). For time evaluation, we tested it on two sizes of images: typical relatively small cropped images of size $1000\times 1000$ pixels and separated TMA cores of size $16{,}000\times 13{,}000$ pixels.

**Figure 6:**
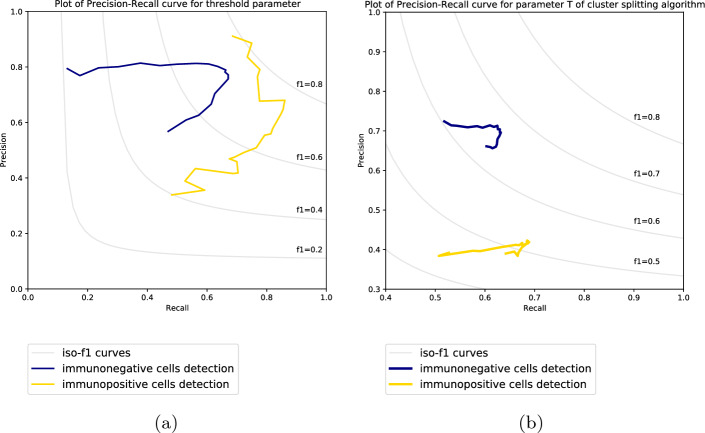
Precision-recall curves for CHISEL’s parameter tuning. Isolines indicate the regions of different F1 scores. (left) The curve is generated by varying the value of threshold parameter of the segmentation algorithm. (right) The curve is generated by varying the value of T parameter of the cluster splitting algorithm.

There are two main parameters of CHISEL that decide about nuclei detection performance. First one is the threshold parameter of the segmentation algorithm that decides about the local relative cut-off level of intensities in processed window and its relevance was established in an earlier study^54^. The other one is the parameter T of the recursive local processing of cluster splitting algorithm that impacts the number of necessary recurrences. Lower values of parameter T works better for heavily packed clusters but the time of processing is increased as presented in previous experiment^58^. To optimize the performance of CHISEL, these tunable parameters were adjusted. We employed twofold cross-validation for tuning the parameters in the experiment. Figure 6 shows the two precision-recall curves. The first curve was generated by varying the value of threshold parameter of the segmentation algorithm (Fig. 6, left), while the other one was constructed by varying the value of T parameter of the recursive local processing of cluster splitting algorithm (Fig. 6, right). The threshold values were empirically chosen to maximize the F1 score on the dataset. As a result the CHISEL is optimized for the resolution of images and the size of nuclei, and it could be successfully used on a new dataset with similar features.

The final part of CHISEL framework, the ANN that classifies the objects, was optimized by analyzing the influence of number of layers, number of neurons, and activation functions on the results of classification. Networks with 1, 2, and 3 hidden layers were tested, nevertheless single hidden layer managed to answer to the problem quite sufficiently. Following the rule of minimal necessary requirement we have kept only one hidden layer. The accuracy of classification was consistently above 97% independent of the number of neurons in the hidden layer, tested in the range from 10 to 20, while dropping with less neurons. Moreover, multiple activation functions were tested and it was fund that they have minimal influence on the result. Finally, this classifier consists of one hidden layer with 20 neurons with sigmoid activation function, and the output layer is a softmax with two output classes: immunopositive and immunonegative nuclei.

The training of ANN was based on typical scheme with 70/15/15 split of examples for training, validation and test subsets. The layers of the network were initialized with Nguyen-Widrow layer initialization function. For training the scaled conjugate gradient backpropagation algorithm was used and cross-entropy function was calculated to evaluate the network’s performance. Training was set for 1000 epochs where one epoch of training is defined as a single presentation of all input vectors to the network. The network (weights and bias) is updated accordingly after each epoch. Concluding, the classification resulted in a mean accuracy of 99.2% which was tested with fivefold cross-validation.

### Comparative visual assessment

We measured the performance of the system on microscopic images from two datasets by comparing their processed results with the annotated ground truth. The performance was validated visually as well as using a previously described evaluation method with PPV, TPR, and F1 score.

**Figure 7:**
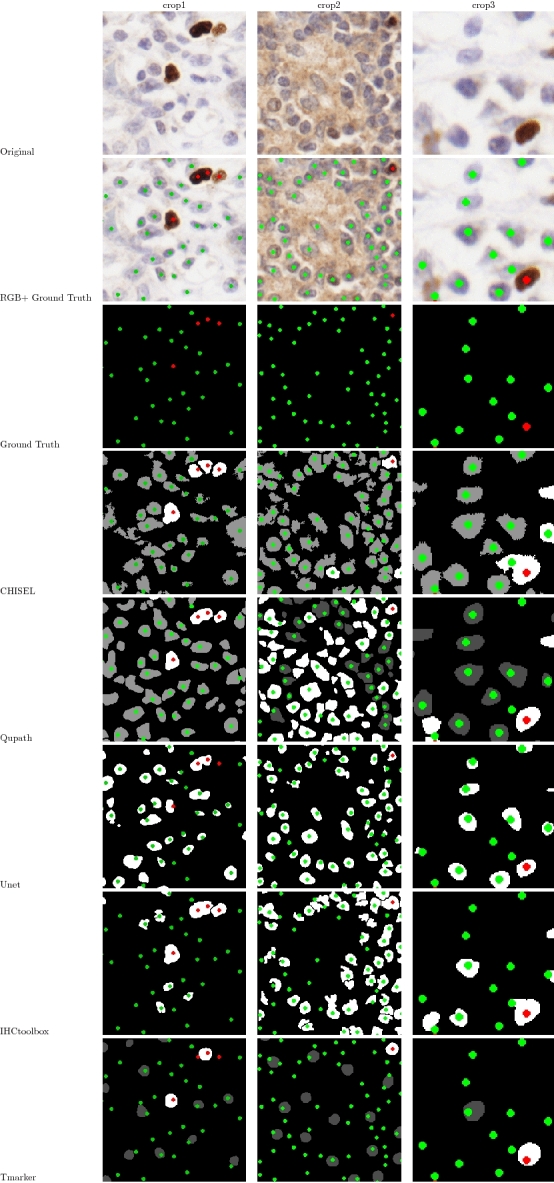
Visual comparison of specific results of five different methods. Ground Truth: green labels = immunonegative nuclei; red labels = immunopositive nuclei. In results: gray area is an object classified as immunonegative; white area is an object classified as immunopositive. The IHCtoolbox and Unet methods allowed only the detection of nuclei, and hence, all the objects are presented as white.

As shown in Fig. 7, the proposed algorithm performs excellent cluster splitting. The immunopositive nuclei were properly divided only by CHISEL (presented in the column “crop1”). In comparison, Tmarker showed multiple missed detections, although its classification seemed correct. The methods Unet and IHCtoolbox provided only detection results (no discrimination between classes), and so all the objects were marked with one (white) color. Conversely, the Qupath method detected too many objects in comparison to the available ground truth.

### Quantitative comparison of the results

The usefulness of the proposed framework was verified by comparing it with the other state-of-the-art software for quantification in histopathology, which are available online, as well as with the DL methods.

**Table 1:** The results of detection and detection followed by classification of CHISEL and other state-of-the-art methods based on the processing of the IISPV dataset.

Method	IISPV dataset results								
	Detection (without classification)			Detection + classification					
	TPR	PPV	F1-score	Immunopositive			Immunonegative		
				TPR	PPV	F1-score	TPR	PPV	F1-score
CHISEL	0.816	0.707	0.748	0.796	0.796	0.767	0.79	0.734	0.749
Qupath	0.906	0.520	0.635	0.961	0.459	0.547	0.737	0.550	0.557
Tmarker	0.265	0.587	0.334	0.479	0.958	0.664	0.232	0.506	0.287
IHCtoolbox	0.541	0.848	0.643						
Unet	0.459	0.724	0.555						
Chen	0.776	0.700	0.724						

The comparison of the proposed framework with the abovementioned methods based on the processing of the IISPV dataset is presented in Table 1. For comparison with Qupath, the expert experienced in this software adjusted manually the processing parameters to achieve the best results. In the case of this dataset, we evaluated the performance of detection as well as the identification of immunopositive and immunonegative nuclei.

In terms of nuclei detection, the highest F1 score was achieved by CHISEL. Qupath topped CHISEL in terms of TPR, but with low PPV its final F1 score was lower. On the contrary, IHCtoolbox had very high PPV but low TPR and therefore had a lower F1 score than CHISEL. The Chen’s DL method was also noteworthy as it showed rather good overall results.

Apart from the proposed method, only Qupath and Tmarker allowed built-in discrimination between the immunopositive and immunonegative nuclei. The results for immunopositive nuclei were very similar to those of detection as described above. However, in the case of immunonegative nuclei, the CHISEL method showed higher values for all metrics.

Table 2 presents the comparison of the proposed framework with other methods based on the Warwick Beta Cell Dataset. The performance of detection of the proposed method was compared to that of the previously mentioned methods. Furthermore, we present the original results of the authors who created this dataset, directly from their published paper^11^. Since these two methods localize only beta cells, we treat them as methods of detection with classification. In the Warwick Beta Cell Dataset, the ground truth was provided as the set of binary masks, corresponding to each image, containing point markers fused from labels made by three experts. Since only markings of beta cell nuclei were provided, the performance of these methods in the detection of objects other than beta cells could not be evaluated. This dataset is quite complex for the detection task. Only CHISEL and IHCtoolbox achieved a TPR higher than 0.5, while all the other methods showed a very poor PPV. Inclusion of classification in the CHISEL framework greatly improved its F1 score as its PPV became the highest. The Lipsym method showed the greatest TPR value, but its lower PPV resulted in a lower F1 score in comparison to the CHISEL method.

**Table 2:** The results of detection and detection followed by classification of CHISEL and other state-of-the-art methods based on the processing of the Warwick Beta Cell Dataset.

Method	WBCD dataset results					
	Detection			Detection + classification		
	TPR	PPV	F1-score	TPR	PPV	F1-score
CHISEL	0.761	0.344	0.449	0.572	0.694	0.615
Qupath	0.463	0.055	0.091	0.427	0.457	0.428
Tmarker	0.112	0.065	0.072	0.038	0.186	0.069
IHCtoolbox	0.615	0.093	0.149			
Unet	0.144	0.066	0.075			
Chen	0.431	0.071	0.112			
Lipsym^a^				0.632	0.606	0.608
LoG^a^				0.472	0.621	0.524

^a^Results directly from publication^11^.

### Discussion of results

The main goal of the CHISEL framework presented in this paper is the automatic quantification of nuclei in IHC-stained tissue samples. Since the main goal of the proposed framework was efficient and precise segmentation of cell nuclei in histopathological sections that were IHC-stained with DAB&H, we used the IISPV dataset of tissue from biopsy of breast cancer patients, in which markers of T cells were used, as our primary data for evaluation. As presented in Table 1, the CHISEL method showed overall best results, as confirmed by its best F1 score. It could be seen that the TPR of Qupath was higher, but its performance was inferior due to poor PPV.

To further establish the relevance of the proposed framework, we evaluated the results obtained with the processing of the Warwick Beta Cell Dataset of DAB&H-stained tissue from postmortem diabetes patients. Working with this dataset required adjusting the framework parameters, but it was rather effortless and not much time-consuming. The objects of interest in Warwick Dataset were similar enough in terms of contrast and stain intensity, although approximately half in size, to ease the adjustment.

As presented in Table 2, our framework showed satisfactory results with this dataset. The initial poor PPV of CHISEL in detection was greatly improved when classification was also considered. This approach achieved the best F1 score among the tested methods, with a slightly more impact on PPV than TPR. In comparison, the Lipsym method (with the second best F1 score) balanced TPR and PPV but the impact was dominant on TPR.

The main issue we encountered while working with Warwick Beta Cell Dataset was the huge interobserver variability between the experts. The comparison of the labels made by the three experts against each other and against the fused ground truth showed that their sensitivity and PPV did not exceed 65%. This proves the difficulty in human interpretation of the images in this dataset. This might also be the reason behind that our and other methods did not exceed the human-level performance.

### CHISEL vs deep learning

The comparison of the proposed workflow with the DL methods indicated an interesting fact. The promised robustness of DL is still unachievable, and in most cases, the models are fitted for processing specific dataset. The DL techniques that seem to entirely overtake the image processing do not perform very well in this scenario. The classification of various sorts of cancer tissue samples can be accomplished with deep neural networks. However, precise quantification of nuclei remains a challenging problem even for DL algorithms^64^. This is mainly due to the lack of publicly available datasets with annotated IHC images. There is plenty of labeled datasets of H&E images, but we have proven in a previous study that a DL model pretrained on H&E images does not outperform our detection method^54^. That study have tried and showed that with very few annotated IHC images it is easier to adjust the parameters of classical image processing workflow rather than training a DL model.

Additionally, one of the major issues with the application of DL method in clinical practice is that clinicians distrust neural network models due to their “black box” constitution and because “the health system is reluctant to completely entrust a machine with a task that a human can do, even if there is substantial time saving”^65^.

Moreover, even inference (in comparison to training) of a complex DL model requires a powerful machine, which is quite costly. This is not always considered a priority resource by medical sector administrators. Of course, it is not the case in well-developed countries but there are still medical stations with limited resources all over the world. Our method has been prepared to fully utilize the available computing power, while being accessible to users with fewer resources. This increases the availability as well as the helpfulness of our implementation.

### Advantages of CHISEL

The overall good performance of CHISEL can be attributed to multiple essential concepts utilized during method development. We have previously established^54^ that the method used for initial segmentation has a high sensitivity for both immunopositive and immunonegative nuclei, while its specificity might not be satisfactory. However, the idea of combining this decent detection algorithm with object classification allows achieving good performance, as indicated by the results presented in Table 1. In addition, the initial detection of nuclei with the subsequent explicit stage of cluster splitting and boundary refinement in CHISEL was proven to improve quantification^57,58^. Furthermore, since the widely popular color deconvolution algorithm might lead to additional inaccuracies in nuclei segmentation^66^, we decided to use HSV color space based on the results of our previous research^54^, on comparing thresholding effectiveness on various types of monochromatic images (separated channels from various color spaces).

Moreover, patch extraction based on the precalculated ROI location and the method of assembling results from multiple overlapping ROIs are designed for the seamless prediction of nuclei in large whole-slide images. This approach facilitates proper segmentation of nuclei near the border of ROI, as any threshold method (and especially adaptive threshold methods used in this framework) does not correctly process the objects that are near the edge of the image. Due to their reduced size and distorted shape, partially visible nuclei near the border of the image can negatively influence the classification process. With our approach this problem is nullified.

The comparison of the proposed schemes in terms of seamless processing revealed similar results, according to their F1 score, with only a slight increase of F1 score in the case of more shifted ROIs as presented in Fig. 5. This might be caused by the experiment settings. In this experiment we primarily focused on the time of processing and overly downsized the ROI (to 250 pixels so that it fits within $1000\times 1000$ images), that causes undersized amount of overlapped areas. This creates a setting where improvement of F1 score is hard to achieve and it does not show the actual value of the proposed schemes. In the case of whole-slide images, the increase in accuracy should be more distinct, but unfortunately we do not possess fully labeled whole-slide images to evaluate the accuracy numerically.

For small images, the basic block processing function (the *blockproc* method that was treated as a benchmark for processing time evaluation) is faster than the proposed schemes. This could be caused by the optimized memory handling of the built-in function. We suppose that simplifying the image handling makes our scheme faster for bigger images. As shown in Fig. 5, the proposed patch extraction scheme speeds up the processing, while maintaining a similar level of accuracy, for bigger images of cores from TMA; this relation is particularly expected to be further improved for WSI. As expected, Scheme A appears to be the fastest solution. While Schemes B and C increase the area of processing by double-checking the borders of primarily selected ROIs, they also increase the processing time. Scheme B seems to balance between the time of processing and the expected increase in accuracy, and hence, it was used in CHISEL realization.

In the proposed framework, images that are too big to fit in the memory are split into smaller ones and processed in batches, complying with the multiprocessing capacity of the used machine. This encourages the scalability of the method; it can be used on a high-end computing machine as well as on a regular personal computer. Thus, this approach enables the use of this framework even in economically less developed countries.

Furthermore, as stated before, CHISEL approach is based on detection of all nuclei in the processed image (with classification than in the later step). This manner of processing is different from majority of software for nuclei quantification but has crucial advantage. This way CHISEL is more suitable for tissue examination where ratio of immunopositive to immunonegative nuclei is a diagnostic factor. CHISEL provides that information with single sweep through the image. Naturally, the typical count of nuclei per area is also easily calculated based on the CHISEL result.

In addition, based on the comparison of the basic nuclei shape features (“Datasets” section) and the final results of our method on both datasets we can assume that CHISEL is quite robust in adapting to variety of images. As there is no universal method that could be applied to every task in digital pathology we give the possibility to adjust the parameters of CHISEL to maximize its performance. Two primary parameters are the threshold of the segmentation algorithm and the T parameter of the recursive local processing of cluster splitting algorithm (optimization of both described in “Optimization” section). The detection is the critical step and it is extremely important to successfully segment all of the nuclei. Adjusting the value of threshold of the segmentation algorithm is mainly based on the contrast between the nuclei and its surroundings. On the other hand, the value of parameter T along with the limit of tested maximum recurrences of the cluster splitting algorithm, that both can be adjusted in CHISEL graphical user interface, is related to the complexity of the clusters present in the analyzed images. Beside these parameters we enabled the possibility to modify the size of ROI and the size of overlap between them. Also the discrimination of small objects (by default lower than 50 pixels) performed in the postprocessing step could be adjusted if the size of objects of interest is smaller than in presented datasets.

### Disadvantages of CHISEL

As with every method, our solution also has drawbacks. The adaptive thresholding algorithm utilized in CHISEL does not perform well if two nuclei with different intensities are clumped together. This happens when the immunopositive and immunonegative nuclei are located very close to each other, as shown in Fig. 7 (column “crop3”). The nucleus with a lighter intensity is often treated as the background in relation to the darker nucleus nearby. In addition, although the cluster splitting algorithm was designed especially for such types of samples, it is ineffective in dividing clusters with small concaves, as presented in Fig. 7 (column “crop2”).

There is no one universal method for processing all of images. Neither classical nor deep learning based solitary method can successfully quantify nuclei in variety of biopsy digital images. The main disadvantage of the classical image processing approach, such as this, is the need to manually adjust the parameters for new data. In CHISEL, we reduced the number of variables to a bare minimum, and in most cases the default values would give decent results. By giving the possibility of adjusting critical parameters to the user we increase the range of assignments this method could be used in.

Another limitation is that this implementation was developed and evaluated in MATLAB. A probable gain could be achieved by implementing the proposed algorithm in a more basic programming language like C++.

## Conclusion

In this study, we developed a system that deals with the complex problem of nuclei quantification in digitalized images of IHC-stained tissue sections. In its current form it is an end-to-end method which segments separate nuclei, focused on achieving best results for DAB&H-stained breast cancer tissue samples, but it could be adjusted for different tasks, as proven by decent results on WBCD. The novelty of this approach results from the proposed seamless segmentation based on the precalculation of ROI location as well as the use of machine learning and recursive local processing to eliminate distorted (inaccurate) outlines, in order to increase the chance of segmenting multiple overlapping and clustered objects.

The method was validated using two labeled datasets which proved the relevance of the achieved results. Based on the comparison of the ground truth with the results of the detected and classified objects, we conclude that the proposed method can achieve better or similar results as the other state-of-the-art methods.

CHISEL has been already used in the scientific community, precisely in the scientific laboratory of the Molecular Biology and Research Section, Hospital de Tortosa Verge de la Cinta, IISPV, URV (Tortosa, Spain). It was used for the quantification of nuclei in DAB&H-stained breast cancer tissue samples in the research. In the future, the system may be further developed and adjusted to the other research problems in pathology.
